# Improving pulse crops as a source of protein, starch and micronutrients

**DOI:** 10.1111/nbu.12399

**Published:** 2019-08-22

**Authors:** G. H. J. Robinson, J. Balk, C. Domoney

**Affiliations:** ^1^ Department of Metabolic Biology John Innes Centre, Norwich Research Park Norwich UK; ^2^ Department of Biological Chemistry John Innes Centre, Norwich Research Park Norwich UK; ^3^ School of Biological Sciences University of East Anglia Norwich Research Park Norwich UK

**Keywords:** biofortification, essential amino acids, favism, legume, resistant starch, sustainability

## Abstract

Pulse crops have been known for a long time to have beneficial nutritional profiles for human diets but have been neglected in terms of cultivation, consumption and scientific research in many parts of the world. Broad dietary shifts will be required if anthropogenic climate change is to be mitigated in the future, and pulse crops should be an important component of this change by providing an environmentally sustainable source of protein, resistant starch and micronutrients. Further enhancement of the nutritional composition of pulse crops could benefit human health, helping to alleviate micronutrient deficiencies and reduce risk of chronic diseases such as type 2 diabetes. This paper reviews current knowledge regarding the nutritional content of pea (*Pisum sativum* L.) and faba bean (*Vicia faba* L.), two major UK pulse crops, and discusses the potential for their genetic improvement.

## Introduction

The Food and Agriculture Organization (FAO) defines pulse crops as legumes harvested solely for their mature dried seed, which is consumed directly as human food or animal feed (FAO 2015). Although widely consumed hundreds of years ago, today the popularity of pulse crops in developed countries is low as a gradual increase in wealth and food supply over time has led to dietary shifts and greater consumption of meat and dairy products (Souza Monteiro *et al*. 2017). Surveys have shown that consumers believe legumes are hard to incorporate into their diets, due to the preparation they require, and that they are also avoided due to the perception that they cause flatulence (Lea *et al*. 2005). Despite their niche status in the UK and other Western countries, pulse crops remain staple foods in the diets of billions of people worldwide, notably in Africa and South Asia, where pigeon pea (*Cajanus cajan* L.) and chickpea (*Cicer arietinum* L.) are the most widely consumed legumes (Amarakoon *et al*. 2012).

**Figure 1:**
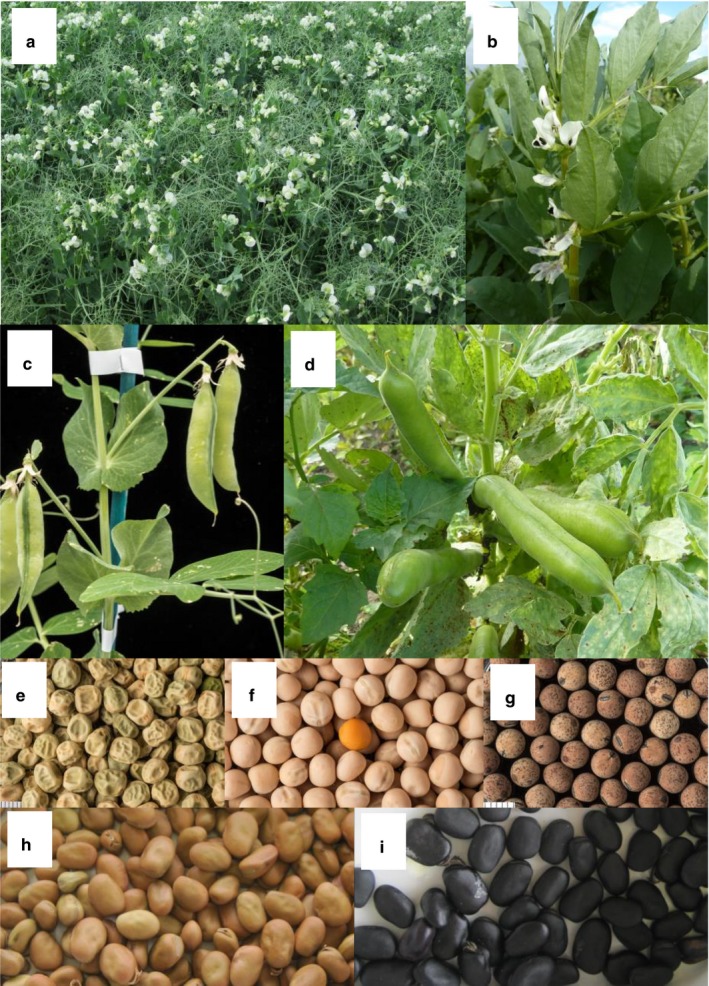
(a) pea and (b) faba bean crops in the field; (c) pea and (d) faba bean immature pods; (e) wrinkled pea seeds, with increased levels of resistant starch; (f) round (smooth) pea seeds of a commercial cultivar (Enigma); (g) seeds of wild pea (*P. elatius*), a species which has provided diversity for a nutritional trait; (h) faba bean seeds with pale testae, preferred for animal feed; (i) faba bean seeds with testae containing high levels of tannins, anti‐nutritional compounds that give a bitter taste. [Colour figure can be viewed at wileyonlinelibrary.com]

Pulses are generally underexploited in agriculture (Foyer *et al*. 2016). During the Green Revolution (1950–1980), the global production of pulses increased by only 60% whereas cereal production almost tripled in the same period (FAO 2019). There has been minimal commercial investment into the primary pulse crops in the UK, pea (*Pisum sativum* L.) and faba bean (*Vicia faba* L.), the latter also known as fava bean or broad bean when immature (Fig. 1). This is due to the reputation among farmers of these crops being low‐yielding, along with the popularity of imported soybean (*Glycine max*) in the ingredients industry (PGRO 2018). The UK is the main pea and faba bean producer in Europe, with total crop yields estimated to be 580 000 tonnes of faba beans and 280 000 tonnes of peas (150 000 tonnes dried and 130 000 tonnes vining) in 2017 (PGRO 2018). Immature pea seeds (vining peas) are used for the frozen and canned vegetable markets, whereas mature dried seeds have a range of uses domestically including in snack products, as ingredients and as mushy peas in the case of marrowfat varieties. A large proportion is exported to the Far East where they are used as snack peas (Pulses UK 2017). Faba bean is one of the world's oldest legume crops (Warsame *et al*. 2018) but, despite once being a staple food, there is now almost no human consumption of the dried bean in the UK, although immature beans may be consumed fresh or frozen. Almost the entirety of the UK faba bean crop is exported or processed for feed, for example, for salmon aquaculture or as feed for ruminant and monogastric livestock (PGRO 2018). The export market for UK‐grown faba bean is dominated by Africa and the Middle East. Egypt alone imports 200 000 tonnes of faba beans annually from the UK (PGRO 2018).

Previous research on pulse crops has focused on improving agronomic traits such as yield, with limited research on the nutritional improvement of these crops (Cooper *et al*. 2017). In addition to being an excellent source of protein, starch and micronutrients, pulses may contain anti‐nutritional compounds that can interfere with the absorption of minerals (Moore *et al*. 2018) and digestion of protein (Clemente *et al*. 2015). Genetic breeding could help to optimise beneficial nutrients while reducing the levels of anti‐nutrients in pulse seeds. This paper reviews the current knowledge of the nutritional composition of pea and faba bean, the potential for genetic improvement, and the recognition of knowledge gaps to be targeted by future research. We also discuss the environmental benefits of growing pulse crops. For a review on the genetic improvement of the nutritional composition of pulses other than pea and faba bean, the reader is referred to Vaz Patto *et al*. 2015). Using pulses as a vehicle for biofortification is also covered in Rehman *et al*. (2019).

## Protein

Pea and faba bean contain relatively high levels of protein compared to cereal crops and therefore could be key contributors to human protein intake, either through consumption in place of animal protein or using pea/faba bean as ingredients, such as in bakery products and snacks. Adequate protein intake is especially important in the older population, as ageing is associated with a decline in muscle mass and a subsequent decrease in quality of life (Stevenson *et al*. 2018). When examining the quality of plant‐based protein sources, the bioavailability and amino acid make‐up of the protein are important factors to consider.

Dry pea seeds contain around 15–30% protein on average, in the form of a limited number of protein types (Table 1). The major fractions of pea seed protein are the water‐insoluble globulins and the water‐soluble albumins. Globulins account for around 50–80% of total pea seed protein and are divided into two groups based on their sedimentation coefficients, the 7S (vicilin and convicilin) and 11S (legumin) fractions. Vicilin is the predominant globulin in pea, but it can vary from around 26–52% of total seed protein, depending on pea genotype (Tzitzikas *et al*. 2006). Albumins represent a much smaller fraction of total seed protein, including lectin and pea albumins 1 and 2 (Le Gall *et al*. 2007).

**Table 1:** Nutrient composition of pea

Fraction	Abundance	Reference
Total protein	15–30% dry weight	Tzitzikas *et al*. (2006); Tao *et al*. (2017)
Globulins	50–80% of protein	Tzitzikas *et al*. (2006)
Vicilin	26–52% of protein	
Legumin	7–25% of protein	
Convicilin	3.9–8.3% of protein	
Albumins	14–42% of protein	Croy *et al*. (1984)
Starch	50% dry weight (round‐seeded)	Bhattacharyya *et al*. (1990)
Resistant starch (% amylose)	30–36% dry weight (wrinkled‐seeded)	
	33–50% of starch (round‐seeded)	
	57–71% of starch (wrinkled‐seeded)	
Minerals		
Iron	45–58 mg/kg (commercial varieties)	Amarakoon *et al*. (2012); Ray *et al*. (2014)
Zinc	39–63 mg/kg (commercial varieties)	Ray *et al*. (2014)
Vitamins		
Thiamin (B_1_)	5.3 ± 0.8 mg/kg	Own experimental data
Riboflavin (B_2_)	0.7 ± 0.1 mg/kg	
Folate (B_9_)	0.54 ± 0.16 mg/kg	

Plants can synthesise all 20 amino acids contained in proteins, but human beings can only make 11 of these, the non‐essential amino acids. The other nine, known as the essential amino acids, must be obtained from the diet. With regard to dietary requirements, legume proteins tend to be low in the essential sulphur amino acids (SAAs) cysteine and methionine, and in tryptophan. Dietary guidelines suggest adults should consume 15 mg/kg of methionine and cysteine and 4 mg/kg of tryptophan per day (United Nations University 2002). Both major globulin proteins in pea contain low concentrations of SAAs, although legumin contains slightly higher levels (1.01–1.78% of protein) (Casey & Short 1981) than vicilin proteins, which largely contain no SAAs (0–0.2%) (Shewry *et al*. 1995; Casey *et al*. 1998). The importance of this lack of SAAs relates to whether or not pea is consumed as part of a balanced diet, as cereal crops provide SAAs lacking in pulses if consumed concurrently and pulses provide lysine, an amino acid which is lacking in cereals (Shewry & Tatham 1999). However, if pea was to become more of a primary protein source, then increases in the levels of essential amino acids would vastly improve its nutritional value. Albumin proteins in pea have been shown to contain higher levels of SAAs than globulins; however, anti‐nutritional properties have been reported for pea albumins, linked to lower digestibility (Vigeolas *et al*. 2008; Clemente *et al*. 2015; Vaz Patto *et al*. 2015). Incorporation of high levels of pea into the diets of nonruminant livestock, such as pigs, has been shown to depress growth, likely due to the low digestibility of albumin proteins such as lectin (Le Gall *et al*. 2007). Trypsin/chymotrypsin inhibitors (TIs) are another class of proteins that affect digestibility (Clemente *et al*. 2015) and are covered in more detail below in the section on anti‐nutrients.

Faba bean seeds can have higher protein concentrations compared to many other legumes, reported as approximately 19–39% of total seed weight depending on genotype (Khan *et al*. 2015; Warsame *et al*. 2018). The amino acid make‐up of faba bean protein is slightly less favourable than that of pea for nutritional purposes, with lower levels of amino acids such as lysine and threonine (Erbersdobler *et al*. 2017). Globulin proteins, predominantly vicilin and legumin, make up 61–78% of total seed protein (Table 2) but, in contrast to pea globulin composition, legumin, rather than vicilin, dominates this fraction in faba bean (Müntz *et al*. 1999; Multari *et al*. 2015). As with other pulse seeds, the low concentrations of SAAs in faba bean storage proteins are notable (Warsame *et al*. 2018).

**Table 2:** Nutrient composition of faba bean

Fraction	Abundance	Reference
Total protein	19–39% dry weight	Khan *et al*. (2015); Warsame *et al*. (2018)
	61–78% of protein	Multari *et al*. (2015); Liu *et al*. (2017)
Vicilin	15–28% of protein	Multari *et al*. (2015)
Legumin	40–55% of protein	Multari *et al*. (2015); Warsame *et al*. (2018)
Albumins	18.4–21.9% of protein	Gasim *et al*. (2015); Khan *et al*. (2015)
Prolamin	3.4–4.3% of protein	Gasim *et al*. (2015)
Glutelin	10.2–12.2% of protein	Gasim *et al*. (2015)
Starch	27–50% dry weight	Kozłowska (2001); Kumar *et al*. (2014); Ivarsson & Neil (2018)
Resistant starch (% amylose)	17–29% of starch	Gunasekera *et al*. (1999)
Minerals		
Iron	23–94 mg/kg (wild and commercial)	Baloch *et al*. (2014); Etemadi *et al*. (2018)
Zinc	10–50 mg/kg (wild and commercial)	Baloch *et al*. (2014); Etemadi *et al*. (2018)

Pulse crops also provide protein isolates that are increasingly being used in the food industry as functional ingredients suitable for vegan diets. The functional properties important for the ingredients industry are solubility, emulsification, foaming and gelling (O'Kane *et al*. 2004). Previously, soybean has dominated the functional ingredients market for plant protein (PGRO 2018). Different protein fractions within crops can convey contrasting functional properties. Vicilin has been reported as a protein that conveys properties such as improved gelling and emulsification ability compared to legumin (Barać *et al*. 2015). This is relevant for a wide range of industrial applications, including the production of gluten‐free bakery items, meat analogues and high‐protein snack bars.

## Starch and sugars

The starch fraction of pulse seeds is a potential source of energy, and pulses are consumed as a staple in South America, Africa and the Indian subcontinent. Starch can be classified as rapidly digestible, slowly digestible or resistant, depending on how easily it is broken down in the gut (Lockyer & Nugent 2017). Starch makes up around 50% of pea seeds by dry weight. The levels of resistant starch in pulse seeds are, on average, 11.6% of total starch when prepared ‘as eaten’, considerably higher than levels observed in cereals (3.2%) and potato (5.7%) (Brighenti *et al*. 1998). This is due to the high amylose–amylopectin ratio in pulse crops, which reduces starch digestibility, desirable for low glycaemic index (Clemente & Olias 2017). Resistant starch cannot be broken down by enzymes in the small intestine and is therefore made available to microflora in the large intestine for fermentation, which results in the production of short‐chain fatty acids (SCFAs), such as acetate and butyrate (Petropoulou *et al*. 2016, 2017). SCFAs are important for the correct functioning of insulin‐producing β‐cells in the human pancreas and help maintain a healthy glucose homeostasis (Petropoulou *et al*. 2016). The ‘slow‐releasing’ nature of resistant starch means spikes in blood glucose are reduced when compared to digestible carbohydrates (Souza Monteiro *et al*. 2017), which decreases the incidence of dysfunction in insulin sensing, the main cause of type 2 diabetes, through improving insulin sensitivity (Johnston *et al*. 2010). Type 2 diabetes is prevalent and ever‐increasing in the UK population, with 5 million people expected to have the condition by 2025 (Petropoulou *et al*. 2016).

Levels of resistant starch are considerably higher in wrinkled‐seeded pea varieties than in round‐seeded lines (Fig. 1), although their total starch is lower (Kozłowska 2001). Pea seeds with lower starch content can contain higher concentrations of some sugars, giving a sweeter taste, which is a desirable trait for garden (vining) peas (Casey *et al*. 1998). The wrinkled‐seeded phenotype in pea was one of the traits studied by Gregor Mendel in the 19^th^ century that led him to propose the basic rules of gene inheritance. More than a century later, it was shown that the phenotype studied by Mendel is due to a naturally occurring mutation in the gene for Starch Branching Enzyme 1 (*SBE1*, Bhattacharyya *et al*. 1990). The mutation causes the starch to be amylose‐rich, with fewer branched amylopectin molecules (Bhattacharyya *et al*. 1990; Rayner *et al*. 2017), giving it increased resistance to digestion (Petropoulou *et al*. 2017). High amylose content, in addition to conveying increased concentrations of resistant starch, is also linked to providing beneficial functional characteristics for the ingredients industry. A high amylose content gives pea starch good thickening and gelling properties, which are important for its use as an ingredient in breads, snacks and soups (Ratnayake *et al*. 2002).

Raffinose oligosaccharides are sugar compounds associated with flatulence (Gawłowska *et al*. 2017). The predominant raffinose oligosaccharides in pea seeds are raffinose, stachyose and verbascose. These compounds are digested by bacterial microflora in the large intestine, sometimes causing flatulence and discomfort in humans, as well as diarrhoea and reduced performance in monogastric livestock. Domesticated pea species and advanced breeding lines have been shown to contain lower levels of raffinose oligosaccharides compared to other groups, suggesting that previous selection by breeders has already favoured lines with lower concentrations of these compounds (Gawłowska *et al*. 2017).

Total starch content is generally estimated to make up around 40–50% of faba bean seeds (Kozłowska 2001), although more recent studies by Kumar *et al*. (2014) and Ivarsson & Neil (2018) suggest that it is closer to 27–40%. A study investigating starch composition in faba bean found resistant starch to range from 8.1 to 15.0% of total starch, and it has been reported that current faba bean varieties have higher glycaemic index values than other pulse crops (Ambigaipalan *et al*. 2011). Silva‐Cristobal *et al*. (2007) examined how the resistant starch content of pickled faba beans changed over time during storage and found it initially made up around 4.1% of total seed weight, decreasing slightly during storage to 3.8%. However, these are the only studies to have examined resistant starch levels in faba bean varieties and it should be noted that contrasting methods for the determination of resistant starch content were used.

## Micronutrients

Pulses are a good source of several micronutrients, in particular iron and some B vitamins, compared to cereal crops and vegetables. The iron content of dry pea seeds was found to range from 45 to 58 mg/kg in commercial cultivars widely grown in North America (Amarakoon *et al*. 2012, 2015; Ray *et al*. 2014). These values are significantly higher than other seed or grain crops (*e.g*. 6.7 mg/kg in white flour derived from wheat) and vegetables (*e.g*. 10 mg/kg in broccoli; 16 mg/kg in spinach). For comparison, the iron content of red meat is 18–36 mg/kg and fortified cereal flours at least 16.5 mg/kg (FSA 2002). Zinc concentrations in commercial pea varieties currently available in the US have been found to range from 39 to 63 mg/kg, depending on the genotype and location grown, higher than zinc levels previously measured in seeds of Canadian pea varieties (27–34 mg/kg) (Ray *et al*. 2014). Pulses are also rich in B vitamins (>15% of reference intake in 80 g), specifically thiamin (vitamin B_1_) and folate (B_9_) (Sierra *et al*. 1998; Jha *et al*. 2015), although there is relatively little research documenting diversity in vitamin B concentrations among pulse crop varieties.

As a source of iron and other micronutrients, pulses have the potential to be useful foods for alleviating nutrient deficiencies which are prevalent throughout the world. An estimated 800 million women and children are affected by iron‐deficiency anaemia globally (WHO 2015), and it is estimated that 17.3% of the world's population is at risk of inadequate zinc intake (Wessells & Brown 2012). Iron deficiency mainly affects haemoglobin levels, causing anaemia, fatigue, a weakened immune system and poor pregnancy outcomes with impaired development in infants due to the role of iron in cell function. Zinc deficiency also affects immune system function and can impair physical growth. Folate deficiency is another prevalent micronutrient deficiency. Folate is essential for DNA synthesis as it is a source of carbon for DNA methylation, and deficiency can lead to macrocytic anaemia and complications in pregnancy such as spina bifida (Irwin *et al*. 2016).

The UK's healthy eating model, the Eatwell Guide, recommends eating more beans and pulses as they are a ‘good alternative to meat because they're naturally very low in fat, and they're high in fibre, protein, vitamins and minerals’. The Eatwell Guide considers 80 g of pulses to count towards one of the recommended 5 A DAY but only once per day (PHE 2018). Pulses can be consumed whole as part of a dish, and flours can be used as an ingredient. In some of the poorest areas of the world, such as Rwanda, the Democratic Republic of the Congo (DR Congo) and Uganda, programmes encouraging the consumption of high‐iron beans have been successful in improving the iron status of young women (Lockyer *et al*. 2018). Pea flour has been trialled as an iron and protein enhancing food ingredient in bread, and it has been shown that bread made with 5% pea flour shows no adverse functional properties (Kamaljit *et al*. 2010). In the UK, wheat flour is currently fortified with iron, calcium and the B vitamin precursors niacin and thiamin ( www.legislation.gov.uk/uksi/1998/141/contents/made), but pulses could provide a natural source of these minerals and vitamins through the incorporation of flour from pulses into wheat flour products. The addition of folic acid to the list of fortificants in wheat flour is currently being considered (BBC 2019).

Iron from plant sources is non‐haem iron, which has lower bioavailability than haem iron obtained from animal sources. Iron bioavailability is mainly governed by plant compounds that either enhance or inhibit uptake (Hurrell & Egli 2010; Lockyer *et al*. 2018). One such compound that is prevalent in pulses is phytic acid; this is discussed in more detail in the next section. A large proportion of the iron in pea seeds is stored as ferritin, which acts as an iron store for the seedling following germination (Briat *et al*. 2010). Plant ferritin is located in plastids, meaning that ferritin–iron is surrounded by several membranes, which can hinder the release of iron during digestion (Moore *et al*. 2018); however, ferritin–iron in purified form has good bioavailability (Lönnerdal *et al*. 2006; Perfecto *et al*. 2018).

As an understudied crop, little is known regarding the micronutrient composition of faba bean (Lombardo *et al*. 2016), with only a handful of studies investigating diversity in mineral nutrients in commercial or wild varieties. Iron concentrations range from 23 to 94 mg/kg in faba bean and have been shown to fluctuate due to environmental conditions (Lombardo *et al*. 2016; Etemadi *et al*. 2018); zinc has been shown to range from around 10 to 50 mg/kg, depending on genotype (Baloch *et al*. 2014; Etemadi *et al*. 2018). The bioavailability of micronutrients in both pea and faba bean is adversely affected by anti‐nutrients, and these compounds are discussed in a later section of this review.

In addition to nutritionally‐relevant micronutrients, faba beans also contain significant levels of 3,4‐dihydroxyphenylalanine, or L‐DOPA (the precursor of dopamine), a compound of high value to the medical industry and of use for the treatment of Parkinson's disease. The consumption of faba beans has been observed to be effective in improving motor performance in Parkinson's disease patients, with 100 g of fresh beans containing 50–100 mg of L‐DOPA (Ramírez‐Moreno *et al*. 2015). The crop therefore presents a real opportunity for molecular pharming of this compound, illustrating its potential benefits in both nutrition and medical arenas.

## Anti‐nutrients

Anti‐nutrients are compounds which can severely affect the bioavailability of nutrients in plant foods (Hurrell & Egli 2010). Even though pulse seeds are nutrient‐rich, the presence of anti‐nutrients in sufficient quantities can reduce nutrient bioavailability and diminish nutritional value. A highly abundant anti‐nutrient in many legume seeds is phytic acid (or phytate). Phytic acid is the major storage form of phosphorus, representing 50–85% of total plant phosphorus in seeds. Ruminants are able to break down phytate through the presence of phytase in their polygastric digestive systems; however, humans and monogastric livestock lack the required enzymes in sufficient quantities (Gupta *et al*. 2015). Because phytate accumulates while seeds mature, immature peas, widely consumed as garden peas or petit pois, have a lower phytate concentration than mature peas but the same concentration of iron–ferritin (Moore *et al*. 2018). It has been shown that the iron: phytate ratio in peas is positively correlated with iron bioavailability and is higher in immature than mature pea seeds. Processing of pulse‐based foods can reduce phytate levels, with phytase enzymes commonly added to achieve this in commercial settings (Gupta *et al*. 2015). Phytate concentrations in faba bean are similar to, or a little higher than, those of pea (Oomah *et al*. 2011), and the majority of studies concerning phytate in this crop have focused on how phytate levels are altered by different processing techniques, rather than through genetic improvement.

Seed protease inhibitors constitute another group of compounds that can reduce the value of pulse seeds within the food and feed industries (Clemente *et al*. 2015). Trypsin/chymotrypsin inhibitors prevent the breakdown of nutritionally valuable proteins in the gut, and their presence means that seeds must be heat‐treated before use (Clemente *et al*. 2015). The most abundant trypsin/chymotrypsin inhibitors in pea seeds are Bowman–Birk inhibitors, a specific class of protease inhibitors discovered more than 70 years ago (Birk 1985). Ingestion of Bowman–Birk inhibitors by rodents and birds causes enlargement of the pancreas and hypersecretion of digestive enzymes. These effects in turn cause a loss of sulphur‐rich endogenous proteins, further exacerbating the issue of the relatively low content of SAAs in pulse crops (Guillamón *et al*. 2008). Trypsin/chymotrypsin inhibitors are also present in faba bean, at levels that are generally lower than in many other pulse crops (Guillamón *et al*. 2008).

Polyphenols and tannins are well known to affect the bioavailability of micronutrients in food in general (Hurrell & Egli 2010) and in pulse crops in particular (Wang *et al*. 1998). Polyphenols are abundant in the testae (seed coats) of pulses and are responsible for the dark colours or spotted patterns of some varieties (Fig. 1). In faba bean, tannins not only reduce protein digestibility but also give a bitter taste. The removal of the testae decreases the levels of polyphenols and removes the bitterness, but this process can be time consuming and thus expensive in the production of food and feed. In a study of Canadian commercial pea varieties, total phenolics were seen to vary between 162 and 325 mg/kg (dry matter). Tannins, which precipitate proteins and make them resistant to digestive enzymes, were found to be barely detectable in all commercial cultivars, but it should be noted that all the cultivars studied had white seed coats (Wang *et al*. 1998). Tannins are referred to as a ‘double‐edged sword’ in biology because, in addition to their anti‐nutritional properties, they have high antioxidant activity (Hagerman *et al*. 1998) and have been suggested to have anti‐HIV activity and anti‐cancer properties, as demonstrated through *in vitro* and *in vivo* studies on rodents (Chung *et al*. 1998; Dahl *et al*. 2012). Anti‐nutritional factors contribute to plant defence against pests and diseases (Rehman *et al*. 2019), meaning that their removal could compromise plant performance in the field. Therefore, both the potential agronomic and health benefits of tannins and other anti‐nutrients, including Bowman–Birk inhibitors (Kennedy *et al*. 1996; Clemente & Olias 2017), must be considered when attempting to reduce their levels through breeding programmes.

The pyrimidine glycosides, vicine and convicine, are anti‐nutrients that have been studied extensively in faba bean (Gutiérrez *et al*. 2006; Crépon *et al*. 2010; Lessire *et al*. 2017); however, their biosynthetic pathways have not yet been elucidated (Khazaei *et al*. 2015). High vicine–convicine levels have a deleterious effect on egg laying, egg quality and red blood cells in laying hens and are therefore not favoured as feed for the poultry industry (Lessire *et al*. 2017). Vicine and convicine also pose a health risk to some humans, causing favism in individuals deficient in glucose‐6‐phosphate dehydrogenase (G6PD). Favism causes haemolysis of erythrocytes and in severe cases can result in the destruction of up to 80% of red blood cells, which is often fatal. G6PD deficiency is common in the Middle East and Mediterranean regions, co‐occurring with high faba bean consumption, resulting in widespread health issues (Crépon *et al*. 2010).

## Pulses as sustainable food sources

In the Western world, protein intake is dominated by meat and dairy. However, the intensive agricultural production of animal protein has devastating effects on the environment through the rearing of livestock and the manufacturing of their feed (Steinfeld *et al*. 2006). The livestock agricultural sector covers around 30% of the ice‐free land on the planet and is responsible for 18% of anthropogenic greenhouse gas emissions, including 9% of emitted carbon dioxide, 35–40% of emitted methane and 65% of emitted nitrous oxide (Steinfeld *et al*. 2006). With the global population set to reach almost 10 billion by 2050, agriculture must be made more sustainable and a large part of this task will require a dietary shift from high meat consumption to more plant‐based foods (Poore & Nemecek 2018). A dietary shift including a reduction in the consumption of unhealthy foods such as red meat, and an increase in the consumption of legumes, nuts, fruit and vegetables would additionally improve human health and wellbeing, and could reduce premature deaths worldwide by 19‐23%  (Lucas & Horton 2019).

Plant‐based diets have risen in popularity in recent years, likely reflecting an increase in concern about the environmental, ethical and health effects of diets dominated by animal derived foods. The UK Vegan Society reported that the number of people following vegan diets quadrupled to over 600 000 between 2014 and 2018 (The Vegan Society 2018), and market research in the US showed that sales of plant‐based products grew by 17% in 2018 (The Good Food Institute 2018). National surveys also indicate that the UK population's consumption of red and processed meat has decreased in the last decade (PHE 2019).

Pulses top the list of sustainable crops for several reasons. As discussed earlier, they are a useful source of carbohydrates, protein and micronutrients. Moreover, pulses grow in a wide range of climates and soil types (Crépon *et al*. 2010) and, most importantly, pulse crops fix atmospheric nitrogen in the soil through symbioses with rhizobial bacteria within their root systems (Curatti & Rubio 2014). This process means their cultivation does not require the application of nitrogen fertilisers, and using pulses in crop rotation systems can reduce fertiliser requirements for the production of non‐legume crops, such as cereals (Nemecek *et al*. 2008; Souza Monteiro *et al*. 2017). Symbiotic rhizobia are estimated to fix 21 000 000 tonnes of nitrogen globally each year, returning 5 000 000–7 000 000 tonnes of nitrogen to the soil. Reducing the volume of nitrogen fertiliser used in agriculture would have significant advantages: the production of fertiliser uses huge amounts of energy and much of this (up to 1% of the global primary energy supply) is effectively wasted as a large proportion of the nitrogen fertiliser applied is lost back to the environment (Curatti & Rubio 2014). Lower fertiliser application also reduces greenhouse gas emissions and the pollution of natural environments through nitrogen runoff into water courses (Barton *et al*. 2014; Foyer *et al*. 2016).

Pea and other pulse crops have been shown to have some of the smallest footprints of all foods and can be considered to be practically carbon neutral (Poore & Nemecek 2018). As a protein source, pulses are considerably less taxing on the environment than animal sources, especially when protein conversion efficiency is considered; animals are inefficient processors of calories as well as protein. The volume of greenhouse gases released by the cultivation of legumes is five to seven times lower per unit area than that of other crops (Stagnari *et al*. 2017). Additionally, tenth‐percentile emissions for dairy beef agriculture are 36 times greater than those of pea (Poore & Nemecek 2018).

## Biofortification of pea and faba bean

Improvement of crops by selection (breeding) is as old as agriculture itself but until recently has been limited to traits of yield, disease resistance, colour and taste. Following the development of methods for molecular analysis, genetic improvement of the protein, starch, vitamin and mineral content of crops can be contemplated. This process is called biofortification, in which the nutritional quality of food crops is improved through agronomic practices, conventional plant breeding or modern biotechnology. Examples of biofortified varieties that are currently grown by farmers are yellow cassava rich in vitamin A in DR Congo and Nigeria; high‐iron beans in DR Congo and Rwanda; and pearl millet with increased iron in India (Lockyer *et al*. 2018). While a genome sequence is not a prerequisite for efficient biofortification, the availability of this greatly facilitates the breeding process. The pea genome is relatively large (~4.45 Gb), and a draft sequence will be available shortly to complement the many genetic resources being developed for this crop (Tayeh *et al*. 2015). The faba bean genome has received more attention in recent years (Webb *et al*. 2016) but has not yet been sequenced due to its size (~13 Gb). Resources such as a multi‐parent population are being developed for genomic studies in faba bean (Khazaei *et al*. 2018).

Pea seed protein composition is complex genetically, with multigene families encoding different proteins (Bourgeois *et al*. 2011), and therefore individual mutations in single genes may have little influence on total protein concentration. However, disrupting the production of lower value proteins, such as lectin and convicilin, has shown the potential for altering pea seed protein composition (Domoney *et al*. (2013).

SAAs are limiting in pulse crops but essential in human diets, and biofortification has proved difficult in breeding and genetic engineering studies on a range of plant species, including maize (*Zea mays)* and *Arabidopsis*, with few examples of success (Galili & Amir 2013; Warsame *et al*. 2018). Although challenging, a fortification method combining the overproduction of free SAAs and the enhancement of SAA‐rich seed proteins has yielded success in narbon bean (*Vicia narbonensis*), a pulse crop and close relative of faba bean (Demidov *et al*. 2003). Demidov *et al*. (2003) used seed‐specific promoters to express a Brazil nut 2S albumin storage protein along with the enzyme aspartate kinase to enhance seed methionine levels by up to 2.4 times compared with wild type.

Genes involved in starch biosynthesis can also exert an effect on seed protein concentration and composition. Mutations at genetic loci in pea, resulting in decreased starch content (Casey *et al*. 1998), also alter legumin: vicilin ratios, total protein concentrations and the relative proportion of the albumin fraction of seed protein (Hughes *et al*. 2001). Studies using chemical mutagenesis have identified multiple loci in pea that alter starch metabolism: *r*,* rb, rug3*,* rug4* and *rug5* (Hylton & Smith 1992; Bogracheva *et al*. 1999). *rug3* encodes plastidial phosphoglucomutase and is of particular interest as mutations at this locus can give rise to almost starchless pea seeds. The most extreme phenotypes have only 1% of the seed dry weight as starch and are extremely wrinkled at maturity (Harrison *et al*. 1998). Genes relating to starch biosynthesis have been well studied in pea, and this provides good potential for the manipulation of pea starch content and composition for various applications. Shen *et al*. (2016) showed that although total starch content is reduced in peas high in protein, their amylose content is significantly higher; this suggests that pea lines could be developed that are good sources of both resistant starch and protein.

Although there does appear to be genetic variation in the concentrations of raffinose oligosaccharides in the pea germplasm (Gawłowska *et al*. 2017), the discovery of mutant lines is required if pea cultivars with very low concentrations of these sugars are to be developed. Drastically reducing levels of raffinose oligosaccharides would prevent issues surrounding flatulence and discomfort and could improve the popularity of pulse crops among consumers. Raffinose synthase is a vital enzyme in raffinose biosynthesis (Peterbauer *et al*. 2002) and is a suitable target in attempts to reduce concentrations of these compounds in pea.

Genetic variation in iron and zinc content of peas (Diapari *et al*. 2015; Demirbaş 2018) suggests that the concentrations of these minerals can be maximised in commercial varieties, as has been achieved for bean (Lockyer *et al*. 2018). Various studies have examined the genetic basis of the iron content in seeds from current germplasm stocks and have had success in finding genetic markers and quantitative trait loci to aid breeding programmes (Diapari *et al*. 2015; Ma *et al*. 2017; Gali *et al*. 2018). Hyperaccumulation mutants, *bronze* (*brz*) and *degenerate leaves (dgl*), have previously been identified in screens of pea mutants and display greatly increased iron uptake. The iron concentration in *dgl* mutant seeds was 163 mg/kg, compared to 65 mg/kg in wild‐type seeds. However, although *brz* plants showed increased iron uptake, there was no increase in seed‐iron content and iron overaccumulated in other parts of the plant, causing phytotoxicity (Kneen *et al*. 1990). Mineral accumulation mutants are otherwise non‐existent in legume species, and a greater understanding of the translocation of minerals within pulse crops is needed. Selenium is an essential trace element for humans and animals (Poblaciones & Rengel 2017), but plants do not have a need for this element (White 2016). Nevertheless, plants – including pea – have been shown to readily take up and accumulate selenium from the soil. Due to the importance of selenium for human and animal health, biofortification programmes have used soil fertilisation or foliar application to increase the concentrations of selenium taken up by plants (Poblaciones & Rengel 2017). This is especially common in areas of the world where soil selenium concentrations are very low, such as Finland.

In addition to enhancing seed micronutrient concentrations, improving the bioavailability of micronutrients could be achieved through breeding to achieve lower levels of anti‐nutritional factors, such as phytate, and enhanced levels of absorption‐promoting compounds, such as xanthophyll, ascorbate and beta‐carotene, which are known to promote iron absorption (Hurrell & Egli 2010; Lockyer *et al*. 2018). Biofortification programmes for the reduction of the anti‐nutrient phytate have already yielded success in non‐legume species, with reduced‐phytate varieties of maize and canola (rapeseed) released into international markets (Garg *et al*. 2018). Additionally, a low‐phytate line of common bean (*Phaseolus vulgaris* L.) has been developed, and the consumption of its beans has been shown to  increase iron absorption in young women (Petry* et al*. 2013). In pea, Warkentin and colleagues (2012) used chemical mutagenesis to produce low‐phytate (*lpa*) pea lines in the cultivar CDC Bronco, in which the phytic acid‐phosphorus content was reduced by 60–90% to 1.0–1.6 mg/g. The *lpa* mutants have similar agronomic characteristics to their progenitor, but the 6% decrease in seed weight was associated with a loss in yield of 8.1–17.6% (Warkentin *et al*. 2012; Shunmugam *et al*. 2014). Iron bioavailability was improved by 50–100% in *lpa* lines compared to controls, in experiments that used simulated digestion and absorption into Caco‐2 cells (Liu *et al*. 2015). The relationship between phytate concentration and iron bioavailability was further supported by a study of Moore *et al*. (2018), which showed that lower phytate levels in immature peas correlated with better iron bioavailability compared to mature peas. Bowman–Birk inhibitors, protease inhibitors that reduce the digestibility of protein, are encoded by two genes (*TI1* and *TI2*) in pea. Screening of pea germplasm collections identified a line of wild pea (John Innes Centre accession JI 262) that had mutations within both genes, conveying drastically lower levels of Bowman–Birk inhibitor activity (Clemente *et al*. 2015) and potential for improved amino acid bioavailability.

Not many genetic improvements have been applied to faba bean, except for the generation of zero‐tannin cultivars. Two recessive mutations, *zt‐1* and *zt‐2*, confer a ‘zero‐tannin’ trait in faba bean (Gutiérrez *et al*. 2008). The zero‐tannin trait in faba bean corresponds with the production of white flowers, but the nature of this morphological marker means that the plant must reach its flowering stage before its phenotype can be determined. Although zero‐tannin lines are beneficial for the inclusion of faba bean in animal feeds, it should also be noted that white‐flowered zero‐tannin lines show poorer performance in the field compared with coloured‐flowered (normal tannin) lines, with lower yields and increased susceptibility to disease in the former (Ivarsson & Neil 2018). There seems to be little variation in vicine (6.6–7.8 mg/g) and convicine (2.5–4.4 mg/g) among commercial faba bean accessions (Ivarsson & Neil 2018); however, a spontaneous mutant containing reduced vicine–convicine content (10‐ to 20‐fold less than wild type) was discovered in 1989 (Duc *et al*. 2004). Subsequent work has focused on, and been successful in, identifying genetic markers for the *vc‐* allele (Gutiérrez *et al*. 2006; Khazaei *et al*. 2015), and although the exact gene conveying reduced concentrations of vicine–convicine is unknown, *vc‐* has already been absorbed widely into breeding programmes.

## Summary and future directions

Pulse crops are currently underutilised and play only a small role in human diets in the UK, despite their high nutritional value with respect to protein, resistant starch and micronutrients, and environmental sustainability as crops. Due to their niche nature, research on these crops has been neglected compared to highly studied UK cereals such as wheat. Despite this, significant progress has been made in multiple areas that will assist with improving the nutritional value of pulse crops, notably the alteration of pea seed protein composition (Domoney *et al*. 2013), the identification of the *sbeI* gene, which increases resistant starch in pea (Bhattacharyya *et al*. 1990), and the removal of anti‐nutrients such as phytate (Warkentin *et al*. 2012), protease inhibitors (Clemente *et al*. 2015) and vicine–convicine (Duc *et al*. 2004).

One issue limiting pulse crop biofortification is that genetic variation in protein composition, starch composition and levels of nutrients and anti‐nutrients has not been explored to its full potential. Investigations into global germplasm resources could prove fruitful for improving many aspects of the nutritional value of pulse crops through genetic variation, which must be discovered in order for it to be used. Genetic studies also have an important role to play if pulse crops are to be biofortified successfully, as the genetic bases of many traits are currently unknown. Additionally, if genetic engineering is used to improve nutritional content, current regulations surrounding genetically modified organisms are likely to hinder the use of biofortified lines in commercial settings. Major challenges and solutions for the nutritional enhancement of pea and faba bean are shown in Table 3.

**Table 3:** Challenges and potential solutions for the nutritional improvement of pea and faba bean

Crop	Challenge	Solution
Pea	Increase concentrations of SAAs	Inactivate genes encoding less desirable proteins
		Explore natural diversity in amino acid metabolism
		Investigate possible biofortification
	Improve levels of resistant starch for health benefits	Manipulation of starch biosynthetic genes and their control
		Identify novel variation in germplasm collections
	Decrease concentrations of raffinose oligosaccharides	Develop raffinose synthase mutants to knockout production of these compounds
	Increase seed concentrations of micronutrients	Investigate variation in wild species and landraces
	Decrease concentrations of phytate	Identify mutant lines with greatly decreased concentrations to improve nutrient bioavailability
	Decrease concentrations of Bowman–Birk inhibitors	Introgress identified mutations conveying low levels of these compounds into commercial varieties
Faba bean	Increase concentrations of SAAs	Further research into faba bean protein make‐up and possible manipulation
	Improve levels of resistant starch for health benefits	Screen germplasm collections for lines high in resistant starch
		Identify starch branching enzyme gene variants to increase resistant starch concentrations
	Increase concentrations of micronutrients	Investigate variation in wild species and landraces
	Decrease concentrations of phytate	Identify natural or derived mutants with low concentrations of phytic acid
	Decrease concentrations of vicine and convicine	Introgress low vicine–convicine trait into commercial cultivars

SAAs, sulphur amino acids.

The broad range of potential uses for UK pulse crops means that there will be no ‘one‐size‐fits‐all’ pea or bean variety that meets the needs for direct human nutrition, animal feed and the ingredients industry. Increased knowledge of variation in pulse crop germplasm and genetic markers for traits should, however, allow varieties with properties desirable for different end‐use applications to be developed. A current significant issue with pulse crops is their low acceptance as a human foodstuff, which may be addressed through genetics, and low popularity among farmers due to concerns regarding crop reliability. When it comes to improving the nutrition of pulse crops, farmers are unlikely to adopt new high‐nutrition varieties if there is a yield penalty as their income depends heavily on the total marketable yield produced. However, the many emerging end‐uses for pulse crops high in protein and other nutrients (*e.g*. pea flour and pea protein isolates) are already creating new and high‐value markets for pulse crops and should encourage farmers to grow these crops and consider quality in addition to crop yield. Public opinion and knowledge of pulse crops has previously hindered their popularity in the Western world, but pulse crops have undergone a recent renaissance due to the emerging popularity of vegan, vegetarian and flexitarian diets and the rapid growth in these markets. If this increase in popularity continues, pulse crops will likely provide wide‐ranging benefits for public health, farming and the environment.

## Conflict of interest

No conflicts of interest have been declared.
